# TCF7L1 regulates cytokine response and neuroendocrine differentiation of prostate cancer

**DOI:** 10.1038/s41389-021-00371-6

**Published:** 2021-11-20

**Authors:** Yu-Ching Wen, Yen-Nien Liu, Hsiu-Lien Yeh, Wei-Hao Chen, Kuo-Ching Jiang, Shian-Ren Lin, Jiaoti Huang, Michael Hsiao, Wei-Yu Chen

**Affiliations:** 1 Department of Urology, Wan Fang Hospital, Taipei Medical University, Taipei, Taiwan; 2 Department of Urology, School of Medicine, College of Medicine, Taipei Medical University, Taipei, Taiwan; 3 TMU Research Center of Urology and Kidney, Taipei Medical University, Taipei, Taiwan; 4 Graduate Institute of Cancer Biology and Drug Discovery, College of Medical Science and Technology, Taipei Medical University, Taipei, Taiwan; 5 General Education Development Center, Hsin Sheng Junior College of Medical Care and Management, Taoyuan, Taiwan; 6 Department of Pathology, Duke University Medical Center, Durham, NC USA; 7 Genomics Research Center, Academia Sinica, Taipei, Taiwan; 8 Department of Pathology, Wan Fang Hospital, Taipei Medical University, Taipei, Taiwan; 9 Department of Pathology, School of Medicine, College of Medicine, Taipei Medical University, Taipei, Taiwan

**Keywords:** Prostate cancer, Cell biology

## Abstract

Neuroendocrine differentiation (NED) is associated with WNT signaling activation and can be significantly observed after failure of androgen-deprivation therapy (ADT) for prostatic adenocarcinomas. Cytokine signaling is stimulated in NED prostate cancer; however, how ADT-upregulated WNT signaling promotes activation of cytokine signaling and contributes to NED of prostate cancer is poorly understood. In this study, we identified ADT-mediated upregulation of transcription factor 7 like 1 (TCF7L1), which increases the cytokine response and enhances NED of prostate cancer through interleukin (IL)-8/C-X-C motif chemokine receptor type 2 (CXCR2) signaling activation. ADT induced the secretion of WNT4 which upon engagement of TCF7L1 in prostate cancer cells, enhanced IL-8 and CXCR2 expressions. TCF7L1 directly binds to the regulatory sequence region of *IL-8* and *CXCR2* through WNT4 activation, thus upregulating IL-8/CXCR2 signaling-driven NED and cell motility. Analysis of prostate tissue samples collected from small-cell neuroendocrine prostate cancer (SCPC) and castration-resistant prostate cancer (CRPC) tumors showed an increased intensity of nuclear TCF7L1 associated with CXCR2. Our results suggest that induction of WNT4/TCF7L1 results in increased NED and malignancy in prostate cancer that is linked to dysregulation of androgen receptor signaling and activation of the IL-8/CXCR2 pathway.

## Introduction

The androgen receptor (AR) signaling pathway was shown to play an essential role in prostate cancer (PCa) [1]. Although targeting the AR is initially clinically effective, most tumors become resistant to androgen-deprivation therapy (ADT) within a few years, due to progression to hormone-refractory or castration-resistant PCa (CRPC) [2]. Reduced AR expression and signaling support increased neuroendocrine (NE) differentiation (NED) of PCa and is associated with the emergence of an aggressive phenotype [3]. Loss of AR signaling in NED PCa (NEPC) leads to decreased levels of serum prostate-specific antigen (PSA), which increases difficulty in early diagnoses [3]. Therefore, identifying consensus molecular mechanisms of NED that are modulated by inhibition of AR signaling is an urgent issue in PCa therapy. In NEPC induced by ADT, several models were proposed [4–6], including WNT [7], SRC [8], and leukemia inhibitory factor (LIF)/interleukin (IL)-6/signal transduction and activator of transcription 3 (STAT3) [9, 10] signaling activation, tumor suppressor (e.g., retinoblastoma 1 (RB1), p53, or phosphatase and tensin homolog (PTEN)) loss of function [11], epigenetic disorders [12], upregulation of transcription factors (e.g., MYCN [13], FOXA2 [14], or ZBTB46 [15]), epithelial-mesenchymal transition (EMT)-stemness crosstalk [16], and hypoxia [17].

It was reported that androgen deprivation promotes an immune response and causes an increase in interleukin (IL)-8 in human PCa cells [18]. IL-8 is thought to be one of the factors contributing to the progression of the castration-resistant state of PCa [19]. IL-8 is specifically expressed by NE tumor cells of human PCa tissues [20]. Upregulation of IL-8 and its receptor, C-X-C chemokine receptor type 2 (CXCR2), and their autocrine actions are critical factors in promoting the progression and metastasis of colon cancer cells [21]. Overexpression of CXCR2 was reported to promote invasion and metastasis of lung adenocarcinomas [22]. Despite IL-8 and CXCR2 having been found to be highly expressed in NE cells of PCa [20], the mechanisms through which upregulation of IL-8/CXCR2 signaling promotes NED and malignant progression of prostate adenocarcinomas post-ADT are not clear.

WNT is a family of signaling proteins that has pivotal roles in neurodevelopmental processes and tumor progression [23]. Activation of the canonical WNT pathway leads to transactivation of target genes, which are involved in the NED of PCa [24]. Inhibition of WNT signaling has the potential to reduce the NED of PCa that drives cancer progression and thus could improve therapeutic outcomes [25]. Nevertheless, activation of AR signaling can repress WNT-mediated transcription induced by androgen in PCa cells [26]. Those findings suggest that inhibition of AR expression might activate the highly upregulated WNT signaling pathway. Therefore, the relationship between the AR and WNT signaling pathway needs to be further explored in order to characterize possible NED mechanisms in PCa after ADT.

Transcription factor (TCF) 7 like 1 (TCF7L1) is a member of the TCF/lymphoid enhancer factor (LEF) family of transcription factors that participate in maintaining stem cell pluripotency [27], homeostasis of skin epithelial tissues [28], determination of cell lineages during stomach formation [29], and vertebrate brain development [30]. It was shown that the growth and invasive capability of highly metastatic breast cancer cells were impaired by ectopic TCF7L1 expression [31]. Overexpression of TCF7L1 was also found to induce the growth of colorectal tumors [32]. TCF7L1 is a candidate oncogene and prognostic marker for skin squamous cell carcinoma (SCC) [33]. Herein, we aimed to understand if TCF7L1 is highly upregulated in prostate tumors after ADT, compared to hormone-naïve prostate patients. We investigated whether there is an association between TCF7L1 overexpression and the NED potential post-ADT in PCa patients. Because activation of IL-8/CXCR2 signaling may increase the induction of CRPC after ADT [34], we showed that TCF7L1 could be the activator of IL-8 and CXCR2 in NED PCa cells through WNT4 activation. We studied the crosstalk mechanism of WNT4/TCF7L1 and IL-8/CXCR2 signals in the NED of PCa to understand the pathogenic mechanism of TCF7L1-dependent activation that predisposes PCa resistance to ADT.

## Results

### TCF7L1 is upregulated in CXCR2^+^ NE cells and correlated with CXCR2 in small-cell NE PCa (SCPC) and CRPC samples

**Fig. 1:**
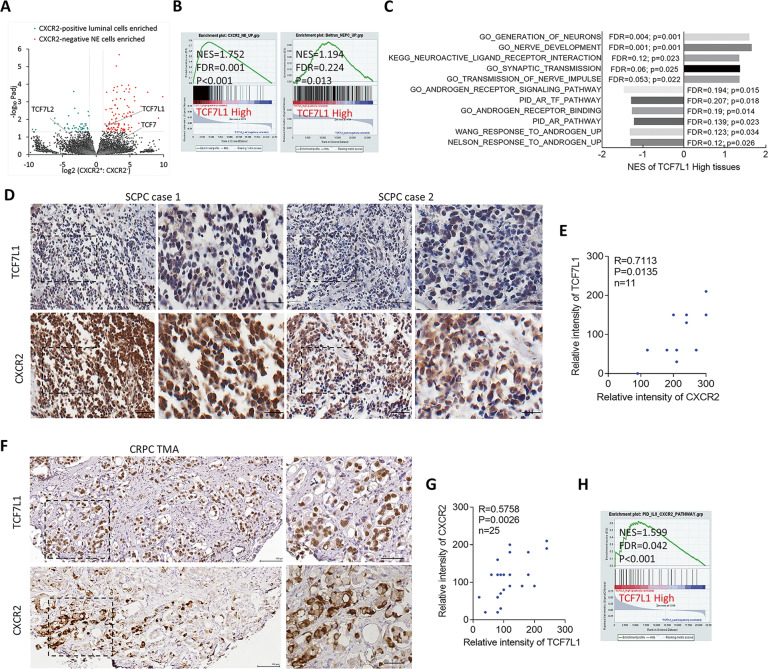
Upregulated CXCR2 is associated with increased TCF7L1 of PCa. Volcano plot of differentially expressed genes between CXCR2^+^ NE and CXCR2^−^ luminal cells analyzed from transcriptome profile data downloaded from GEO (GSE114326). *TCF7L1* is a gene which is significantly upregulated in the population of CXCR2^+^ NE-enriched cells. **B** GSEA showing that higher TCF7L1 expression was positively associated with the CXCR2^+^ NE-responsive signature [36] and NE-responsive signature [38] in TCGA PCa dataset. NES normalized enrichment score, FDR false discovery rate. **C** GSEAs of TCGA PCa dataset showing that higher TCF7L1 expression was positively associated with neuronal development (GO and KEGG) and negatively associated with androgen-responsive signaling (GO, PID, Wang [39], and Nelson [40] gene signatures). **D**, **E** Images and intensity of consecutive tissue sections stained with TCF7L1 and CXCR2 in SCPC samples collected from Taipei Medical University-Wan Fang Hospital. *n* = 11. Scale bars, 100 μm. The **s**ignificance of the positive correlation among the intensities of TCF7L1 and CXCR2 was determined by two-tailed Pearson correlation XY analyses in GraphPad Prism. *R*, correlation coefficient, *P*, *p* (two-tailed) value of SCPC samples. **F**, **G** Images and intensity of consecutive tissue sections stained for TCF7L1 and CXCR2 in a CRPC TMA from the Duke University School of Medicine. Scale bars, 100 μm. Higher TCF7L1 expression was positively associated with higher CXCR2-expressing PCa samples as observed in the CRPC TMA (*n* = 25). The significance of the positive correlation among the intensities of TCF7L1 and CXCR2 was determined by two-tailed Pearson correlation XY analyses in GraphPad Prism. *R*, correlation coefficient, *P*, *p* (two-tailed) value. **H** GSEA of TCGA PCa dataset showing that enrichment of TCF7L1 expression was positively associated with an upregulated IL-8/CXCR2 responsive signature (PID).

As to the association between WNT signaling and CXCR2, it is known that CXCR2 may participate in regulating the lineage commitment of human pluripotent stem cells by activating the β-catenin pathway [35]; however, the mechanism by which CXCR2 regulates NED of PCa via WNT signaling is not clear. Although CXCR2 is highly expressed in NE cells of PCa [20], it is still unclear how WNT signaling upregulates CXCR2 and promotes NED of PCa after ADT. We analyzed RNA-sequencing (RNA-Seq) data (GSE114326) expression in a set of prostatic tumor samples collected from CXCR2^+^ NE cells compared to CXCR2^−^ luminal cells [36]. We focused on the TCF family because we previously showed that TCF transcription factors may promote the malignant progression of PCa after ADT and induce treatment resistance [37], but their NED function is unknown. Unexpectedly, we observed that TCF7L1, a member of the TCF family, was significantly upregulated in CXCR2^+^ NE cells compared to TCF7 and TCF7L2 (Fig. 1A, Supplementary Fig. S1A). In PCa cells, the full-length TCF7, TCF7L1, and TCF7L2 complementary (c)DNAs were transiently transfected into LNCaP cells and analyzed for CXCR2, IL-8, and NE markers (chromogranin A (CHGA), chromogranin B (CHGB), synaptophysin (SYP), and enolase 2 (ENO2)). We found that overexpression of TCF7L1 significantly increased expressions of CXCR2, IL-8, and NE markers, but TCF7 and TCF7L2 only partially increased the expression of IL-8 or CXCR2, but not the expressions of NE markers (Supplementary Fig. S1B–D). A geneset enrichment analysis (GSEA) confirmed that TCF7L1 was highly expressed in PCa tissues with upregulated CXCR2^+^ NE-responsive gene signatures [36] and NE-responsive gene signatures [38] in The Cancer Genome Atlas (TCGA) PCa dataset (Fig. 1B). Furthermore, TCF7L1 upregulation was positively associated with the gene signature of neural development (gene ontology (GO) and kyoto encyclopedia of genes and genomes (KEGG)) and negatively associated with the gene signature of the androgen response (GO, pathway interaction database (PID), Wang [39], and Nelson [40]) as confirmed by a GSEA from TCGA PCa database (Fig. 1C), suggesting that TCF7L1 upregulation after ADT is involved in NED progression. NE cells were shown to accumulate during PCa progression after ADT through interfering with NED [41, 42], and they undergo progression into advanced PCa as a result of SCPC [43]. We sought to validate the positive correlation between CXCR2 and TCF7L1 in advanced PCa tissue samples. We measured TCF7L1 and CXCR2 protein levels in SCPC samples collected from Taipei Medical University-Wan Fang Hospital (Taipei, Taiwan) by immunohistochemical (IHC) staining using consecutive tissue sections, and found that there was a positive relationship between TCF7L1 and CXCR2 (Fig. 1D, E). We next analyzed a CRPC tissue microarray (TMA) collected by the Department of Pathology, Duke University School of Medicine (Durham, NC, USA), and we found a positive association between TCF7L1 and CXCR2 in CRPC samples (Fig. 1F, G). We further validated TCF7L1 expression with gene signatures reflecting IL-8/CXCR2 signaling components in TCGA PCa dataset and found that tissues expressing high levels of TCF7L1 were robustly associated with a gene signature of upregulated IL-8/CXCR2 responsiveness (PID, Fig. 1H). This finding suggests that the NED function is regulated by IL-8/CXCR2 and is connected to upregulation of TCF7L1 in PCa after ADT.

### Activation of IL-8/CXCR2 signaling is upregulated by TCF7L1

**Fig. 2:**
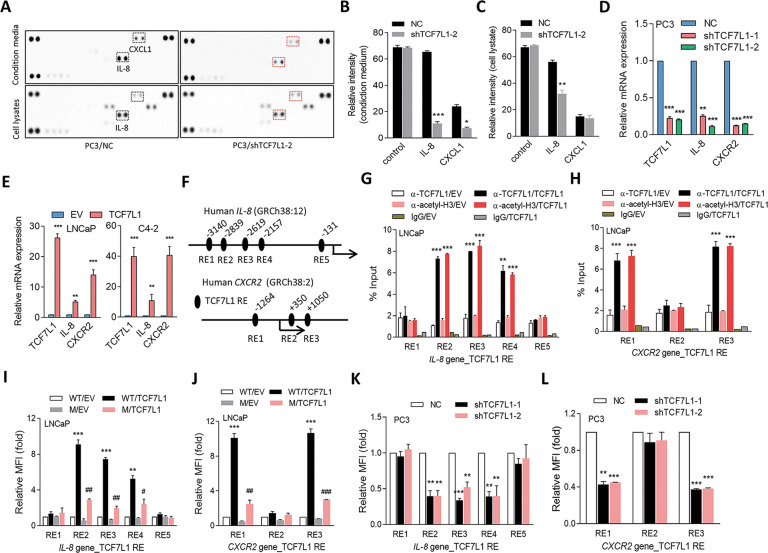
TCF7L1 upregulates IL-8 and CXCR2 through directly binding to the regulatory sequence of *IL-8* and *CXCR2*. **A** Cytokine and chemokine array analysis of PC3 cells stably expressing a nontarget control (NC) or TCF7L1 shRNA vector. Conditioned medium was collected from the supernatant and centrifuged. **B**, **C** Intensity of conditioned medium (**B**) and whole-cell lysates (**C**) in the cytokine and chemokine array analysis from **A**. **D** RT-qPCR showing TCF7L1, IL-8, and CXCR2 mRNA levels in PC3 cells stably expressing the NC or TCF7L1 shRNA vector. **E** Relative mRNA levels of TCF7L1, IL-8, and CXCR2 in LNCaP and C4-2 cells stably expressing an empty vector (EV) or a TCF7L1 expression vector, by an RT-qPCR. Data from the quantification of mRNA are presented as the mean ± SEM; *n* = 3 per group. *p < 0.05, **p < 0.01; by a two-way ANOVA. * vs. the EV. **F** Schematic of the predicted TCF7L1 responsive element (RE) of human *IL-8* and *CXCR2* regulatory sequences. **G**, **H** ChIP assays of LNCaP cells expressing the EV or TCF7L1 cDNA vector with antibodies against TCF7L1 and acetyl-H3 by a pair of primers recognizing indicated TCF7L1-RE sites on *IL-8* (**G**) and *CXCR2* (**H**) regulatory sequences. Enrichment is given as a percentage of the total input and then normalized to IgG. * vs. the EV. **I**, **J** Relative mean fluorescent intensities (MFIs) of wild-type (W) and mutant (M) *IL-8* and *CXCR2* green fluorescent protein (GFP)-reporters in LNCaP cells stably transfected with the EV or TCF7L1 expression vector. **K**, **L** Relative MFIs of wild-type *IL-8* and *CXCR2* GFP-reporters in PC3 cells stably transfected with an NC or TCF7L1 shRNA vector. Quantification of mRNA, ChIP data, and MFIs is presented as the mean ± SEM from three independent experiments. Significance was determined by Student’s *t*-test. *p < 0.05, **p < 0.01, ***p < 0.001.

Despite a WNT-stimulated cytokine response in the microenvironment being required to maintain aggressiveness and metastatic progression in several types of cancer [44], there is little knowledge about the upregulation of TCF7L1 associated with cytokine responses in PCa. We validated our hypotheses by a human cytokine and chemokine protein array analysis in PC3 cells with TCF7L1-knockdown (KD). We identified that a significant reduction in IL-8 was associated with TCF7L1-KD based on both cellular and secreted levels (Fig. 2A–C). We confirmed that TCF7L1-KD significantly decreased messenger (m)RNA expressions of IL-8 and CXCR2 in AR-negative PC3 cells (Fig. 2D). We stably overexpressed TCF7L1 in AR-positive LNCaP and C4-2 cells, and found that IL-8 and CXCR2 mRNAs were upregulated according to a real-time reverse-transcription quantitative polymerase chain reaction (RT-qPCR) analysis (Fig. 2E). In order to understand the relationship between TCF7L1 and CXCR2, we downloaded chromatin immunoprecipitation (ChIP)-sequence data from the Gene Expression Omnibus (GEO) (GSE80331) based on an Illumina HiSeq analysis and analyzed a putative TCF7L1-binding site located in the *CXCR2* regulatory sequence. We found that there was a possible consensus TCF7L1-binding element in the *CXCR2* regulatory sequence located around chr 2:218,989,000 to 218,990,000 (Supplementary Fig. S2A). Next, we looked for sequences resembling the TCF7L1 response element in the putative *IL-8* and *CXCR2* regulatory sequence regions and respectively found five and three candidate binding elements for TCF7L1 in the *IL-8* and *CXCR2* regulatory sequences (Fig. 2F). To test whether TCF7L1 directly binds to the regulatory sequence of *IL-8* and *CXCR2*, we performed ChIP assays and used a qPCR to determine how much chromatin of *IL-8* and *CXCR2* was perceptible by an anti-TCF7L1 antibody vs. a positive control anti-acetyl-H3 antibody, from nuclear extracts of LNCaP cells stably expressing an empty vector (EV) or TCF7L1 cDNA vector. We found significantly increased TCF7L1 binding at the putative TCF7L1-binding site on the responsive element (RE) 2, RE3, and RE4 sites of the *IL-8* gene and on the RE1 and RE3 sites of the *CXCR2* gene in cells with TCF7L1 expression (Fig. 2G, H). We performed reporter assays with a DNA construct containing individual wild-type (WT) and a mutant of the putative TCF7L1 response elements from the *IL-8* and *CXCR2* regulatory sequences (Supplementary Fig. S2B). We found that LNCaP cells overexpressing TCF7L1 had significantly increased reporter gene activities at the RE2, RE3, and RE4 sites on the *IL-8* gene and the RE1 and RE3 sites on the *CXCR2* gene compared to EV-transfected cells (Fig. 2I, J). Nevertheless, decreased reporter activity was seen in cells transfected with the reporter construct containing TCF7L1-RE mutants regardless of TCF7L1 overexpression (Fig. 2I, J). Moreover, decreased *IL-8* and *CXCR2* reporter construct activities from the RE2, RE3, and RE4 sites on the *IL-8* gene and from the RE1 and RE3 sites on the *CXCR2* gene were detected in PC3 cells with TCF7L1-KD (Fig. 2K, L). These data are consistent with a mechanism whereby TCF7L1 promotes *IL-8* and *CXCR2* transcription by direct physical interactions between TCF7L1 and the regulatory sequences of *IL-8* and *CXCR2*.

### Suppression of AR signaling upregulates TCF7L1, which is associated with IL-8/CXCR2-driven NED PCa

**Fig. 3:**
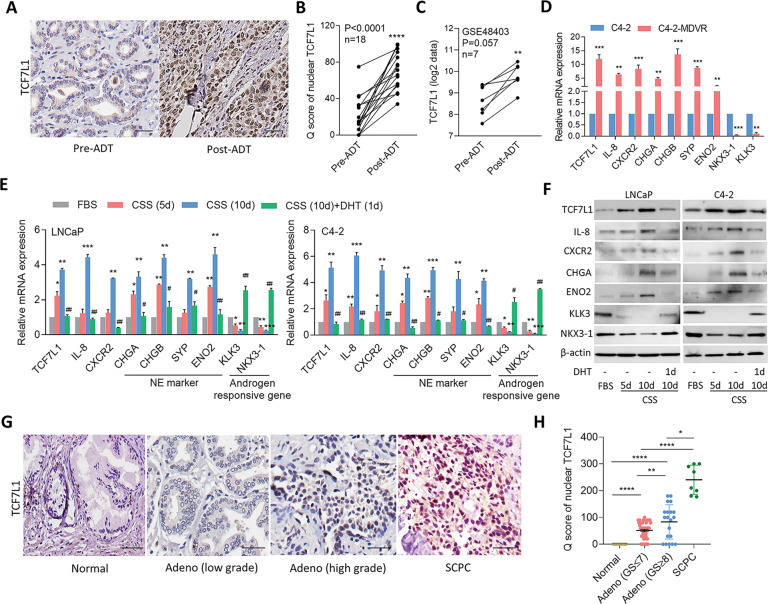
ADT induced TCF7L1 associated with NED of PCa. **A**, **B** IHC staining (**A**) and analysis (**B**) of nuclear TCF7L1 in PCa tissue sections from patients before and after ADT. The 18 samples were collected from Taipei Medical University-Wan Fang Hospital. Scale bars, 100 µm. Statistical analysis was performed using a two-tailed Student’s *t*-test. ****p < 0.0001. **C** Expressions of TCF7L1 in paired PCa samples pre- and post-ADT from the GSE48403 dataset. **D** Relative mRNA levels of TCF7L1, NE markers, and androgen-responsive markers in C4-2 and C4-2-MDVR cells, by an RT-qPCR. Data from quantification of mRNA are presented as the mean ± SEM, *n* = 3 per group. *p < 0.05, **p < 0.01, ***p < 0.001; by a two-way ANOVA. **E** Relative mRNA levels of TCF7L1, NE markers, and androgen-responsive markers in LNCaP and C4-2 cells cultured in CSS-containing medium for 5 and 10 days and then treated with 10 nM DHT for 24 h. * vs. FBS; ^#^ vs. CSS (10 days). **F** Western blot assays of TCF7L1, IL-8, CXCR2, CHGA, ENO2, KLK3, and NKX3-1 in LNCaP and C4-2 cells after treatment with CSS-containing medium for 5 and 10 days and then treatment with DHT for 24 h. **G**, **H** Images (**G**) and relative intensities (**H**) of TCF7L1-positive staining in prostate cancer TMA sections containing normal tissues (*n* = 14), adenocarcinomas with a Gleason score of ≤7 (*n* = 57), adenocarcinomas with a Gleason score of ≥8 (*n* = 20), and SCPC (*n* = 8) from the Duke University School of Medicine by a Q-score analysis. Scale bars, 100 μm. *p < 0.05; **p < 0.01; ****p < 0.0001. Significance of the relative intensities was determined using a two-tailed Student’s *t*-test, and comparisons are indicated as a solid line between the two columns.

Since ADT is one of the main causes of NED [3], we next determined whether upregulation of TCF7L1 was mediated by ADT. We collected PCa tissues from the same PCa patients before and after ADT in Taipei Medical University-Wan Fang Hospital (Taipei, Taiwan). IHC results showed that TCF7L1 levels were higher in PCa patients after ADT compared to the same patients before ADT (Fig. 3A, B). We validated mRNA expression levels of *TCF7L1* in an RNA-Seq dataset (GSE48403) in paired PCa samples pre- and post-ADT, and found that post-ADT patients had increased TCF7L1 expression in the GSE48403 dataset (Fig. 3C), consistent with our data. Moreover, overexpression of TCF7L1 was observed in an AR antagonist-resistant cell line (C4-2-MDVR, which are C4-2 cells exposed to long-term treatment with MDV3100), and that had increased NE marker and decreased androgen-responsive gene expressions compared to parental C4-2 cells (Fig. 3D). C4-2-MDVR cells also showed increased IL-8 and CXCR2 mRNA expressions (Fig. 3D), suggesting that inhibition of AR signaling might upregulate IL-8 and CXCR2 expressions. To mimic an androgen-deprivation condition, AR-positive LNCaP and C4-2 cells were cultured with charcoal-stripped serum (CSS)-containing medium and further responded to activate AR signaling by AR ligand dihydrotestosterone (DHT) treatment. Notably, cells treated with CSS-containing medium exhibited increased mRNA and protein levels of TCF7L1, IL-8, CXCR2, and NE markers, and decreased levels of androgen-responsive markers compared to cells treated with fetal bovine serum (FBS)-containing medium (Fig. 3E, F). Importantly, TCF7L1, IL-8, CXCR2, and NE markers decreased, and androgen-responsive markers increased in DHT-treated cells (Fig. 3E, F), which supports TCF7L1/IL-8/CXCR2 upregulating NED function in PCa cells as observed post-ADT. To determine the tumorigenic role of TCF7L1 in PCa, we used a TMA obtained from the Department of Pathology at Duke University School of Medicine (Durham, NC, USA), which was comprised of normal and PCa-progressed tumor tissues. Interestingly, high-grade tumors had moderately high TCF7L1 expression compared to low-grade and normal tissues (Fig. 3G, H). We also analyzed eight SCPC samples, and found that most of those cases had higher TCF7L1 expression (Fig. 3G, H), consistent with samples collected from Taipei Medical University-Wan Fang Hospital (Taipei, Taiwan). These results supported upregulation of TCF7L1 promoting progression of NED in PCa.

### TCF7L1 promotes the NED of PCa cells

**Fig. 4:**
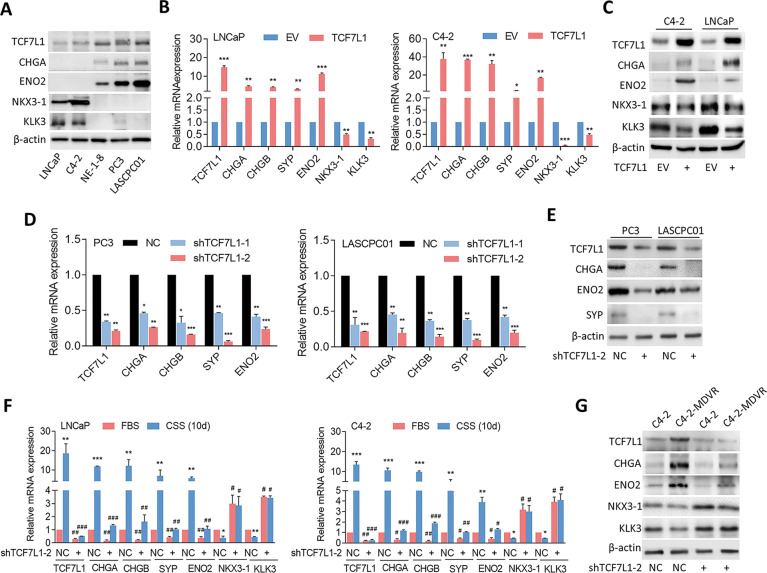
ADT-upregulated TCF7L1 promotes the NED of PCa. **A** TCF7L1, CHGA, ENO2, NKX3-1, and KLK3 protein levels in various PCa cell lines as determined by immunoblotting. **B** RT-qPCR showing mRNA levels of TCF7L1, NE markers (CHGA, CHGB, SYP, and ENO2), and androgen-responsive genes (NKX3-1 and KLK3) in LNCaP and C4-2 cells following stable transfection with an empty vector (EV) or a TCF7L1 cDNA vector. *p < 0.05, **p < 0.01, ***p < 0.001. **C** TCF7L1, CHGA, ENO2, NKX3-1, and KLK3 protein levels in C4-2 and LNCaP cells following stable transfection with an EV or TCF7L1 expression vector, by immunoblotting. **D** mRNA levels of TCF7L1 and NE markers in PC3 and LASCPC01 cells following stable transfection with nontarget control (NC) or TCF7L1 shRNA vectors, by an RT-qPCR analysis. * vs. the NC. **E** TCF7L1, CHGA, ENO2, and SYP protein levels in PC3 and LASCPC01 cells expressing the NC or TCF7L1 shRNA. **F** mRNA levels of TCF7L1, NE markers, and androgen-responsive genes in LNCaP and C4-2 cells expressing the NC or TCF7L1 shRNA following treatment of cells with FBS-containing or CSS-containing medium for 10 days, by an RT-qPCR analysis. * vs. FBS; ^#^ vs. the NC. Data from the quantification of mRNA are presented as the mean ± SEM, *n* = 3 per group; by a two-way ANOVA. **G** Immunoblots showing TCF7L1, CH**G**A, ENO2, NKX3-1, and KLK3 protein levels in C4-2 and C4-2-MDVR cells expressing the NC or TCF7L1 shRNA.

Next, we investigated the functional role of TCF7L1 in the progression of NED. We checked protein expression levels of TCF7L1, NE markers (CHGA and ENO2), and androgen-responsive genes (NKX3-1 and KLK3) in a panel of PCa cell lines. We found that AR-suppressed PC3 cells, the NE-like NE-1-8 cell line [45] (LNCaP cells with long-term androgen withdrawal), and an SCPC-like LASCPC01 cell line [13] exhibited higher expressions of TCF7L1 and NE markers and lower expressions of androgen-responsive genes than did the AR-positive LNCaP and C4-2 prostate adenocarcinoma cell lines (Fig. 4A). To determine if TCF7L1 overexpression can induce the NED of AR-positive cells, we generated LNCaP and C4-2 cells that stably expressed TCF7L1 cDNA and found that the forced expression of TCF7L1 significantly increased mRNA and protein levels of NE markers, and reduced androgen-responsive gene expressions (Fig. 4B, C). In addition, decreased mRNA and protein levels of TCF7L1 and NE markers were found in PC3 and LASCPC01 cells exposed to the TCF7L1 short hairpin (sh)RNA vector compared to a nontarget control (NC) shRNA (Fig. 4D, E). We further knocked down TCF7L1 in AR-positive LNCaP and C4-2 cells and treated those cells with CSS-containing medium to mimic ADT conditions. Results demonstrated that AR-positive cells treated with CSS-containing medium had increased NE markers; however, TCF7L1-KD cells that underwent ADT had decreased expressions of TCF7L1 and NE markers, and constitutively expressed androgen-responsive genes (Fig. 4F). Moreover, MDV3100-resistant cells had increased NE marker and decreased androgen-responsive gene expressions, whereas TCF7L1-KD in C4-2-MDVR cells significantly decreased NE markers and increased levels of androgen-responsive genes (Fig. 4G). These results support TCF7L1 expression being upregulated after AR signaling inhibition, and its overexpression is involved in the NED of PCa cells.

### ADT-resistance increases TCF7L1 and upregulates NED through increased WNT4

**Fig. 5:**
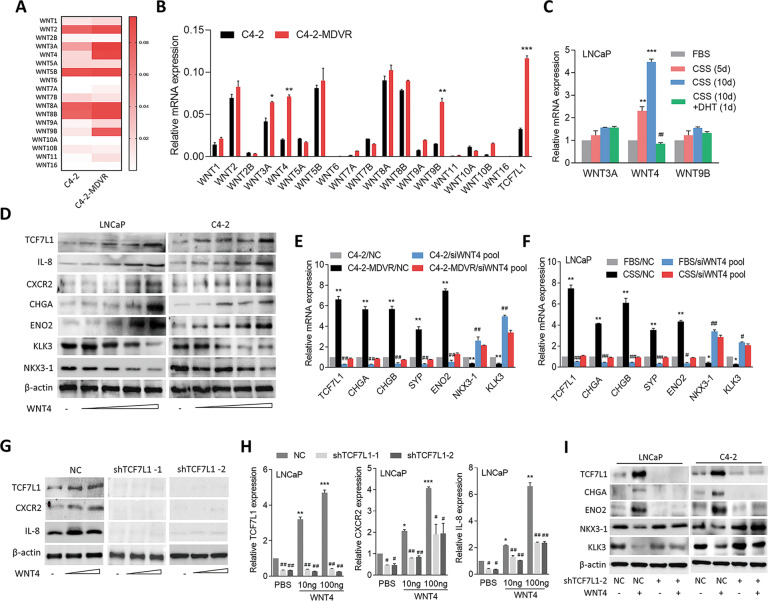
ADT-resistance induced-TCF7L1/IL-8/CXCR2 upregulates NED through increased WNT4. **A** WNT ligand mRNAs as determined by a human RT^2^ Profiler WNT signaling PCR Array analysis of C4-2-MDVR and C4-2 cells. The intensity was determined by the multiple of change of C4-2-MDVR/C4-2 as the magnitude of log2-transformed data from each mRNA level. **B** WNT ligands and TCF7L1 mRNAs in C4-2 and C4-2-MDVR cells, by an RT-qPCR. *p < 0.05, **p < 0.01, ***p < 0.001. **C** RT-qPCR showing WNT3A, WNT4, and WNT9A mRNA levels in LNCaP cells after treatment with CSS-containing medium for 5 or 10 days and subsequent treatment with 10 nM DHT on day 10 for 1 day. **D** Immunoblots showing TCF7L1, IL-8, CXCR2, CHGA, ENO2, KLK3, and NKX3-1 protein levels in LNCaP and C4-2 cells following increased WNT4 protein treatment for 48 h. **E** RT-qPCR showing TCF7L1, NE markers (CHGA, CHGB, SYP, and ENO2), and androgen-responsive genes (NKX3-1 and KLK3) mRNA levels in C4-2 and C4-2-MDVR cells following WNT4-knockdown by siWNT4 SMARTpool. **F** TCF7L1, NE marker, and androgen-responsive gene mRNA levels in LNCaP cells following WNT4-knockdown by siWNT4 SMARTpool in 10% FBS-containing medium or CSS-containing medium for 10 days. **G** Protein levels of TCF7L1, CXCR2, and IL-8 in PC3 cells expressing a nontarget control (NC) or TCF7L1 shRNA vector following increased WNT4 protein treatment for 48 h. **H** mRNA levels of TCF7L1, CXCR2, and IL-8 in LNCaP cells expressing the NC or TCF7L1 shRNA vector following increased WNT4 protein treatment for 48 h, by an RT-qPCR analysis. * vs. PBS; ^#^ vs. the NC. Data from the quantification of mRNA are presented as the mean ± SEM, *n* = 3 per group; by a two-way ANOVA. **I** TCF7L1, CHGA, ENO2, NKX3-1, and KLK3 protein levels in LNCaP and C4-2 cells expressing the NC or TCF7L1 shRNA vector following 100 ng/ml WNT4 protein treatment for 48 h as determined by immunoblotting.

To determine the specific WNT ligand involved in ADT resistance of PCa cells, we analyzed expression profiles of WNT signaling pathway-related genes in MDV3100-resistant C4-2 cells and parental C4-2 cells via an RT [18] profiler PCR array analysis. We found that upregulation of WNT3A, WNT4, and WNT9B mRNAs was enriched in C4-2-MDVR cells compared to parental C4-2 cells (Fig. 5A). The RT-qPCR analysis confirmed that significant increases in WNT3A, WNT4, and WNT9B mRNAs in C4-2-MDVR cells were associated with TCF7L1 compared to parental C4-2 cells (Fig. 5B). In order to test the association of the upregulation of WNT3A, WNT4, or WNT9B with TCF7L1 expression and AR signal inactivation, we examined expressions of WNT3A, WNT4, and WNT9B in AR-positive LNCaP cells in response to ADT. We found that WNT4, but not WNT3A or WNT9B, was upregulated after androgen withdrawal (CSS treatment) and was downregulated in the presence of DHT (Fig. 5C), suggesting that WNT4 overexpression was linked to AR signaling inactivation in response to ADT. Treatment of LNCaP and C4-2 cells with the WNT4 recombinant protein led to a dose-dependent increase in TCF7L1, which was related to increased expressions of IL-8, CXCR2, and NE markers and decreased expressions of androgen-responsive genes (Fig. 5D). Moreover, we examined expressions of IL-8, CXCR2, and NE markers in cells treated with WNT4 protein after pretreatment with the porcupine inhibitor, IWP-O1. It is known that IWP-O1 can prevent secretion of the WNT protein [46]. Our results indicated that IWP-O1 downregulated expressions of IL-8, CXCR2, and NE markers. We also noticed that although cells are treated with IWP-O1, WNT4 can still increase the expression of IL-8, CXCR2, and NE markers compared to treatment with IWP-O1 alone (Supplementary Fig. S1E). These results support an association between activated TCF7L1 and the IL-8/CXCR2 signaling pathway through WNT4 upregulation. Although MDV3100-resistant cells showed induction of TCF7L1 and NE markers and reductions of androgen-responsive genes, we found that when WNT4 was knocked down, MDV3100-resistant cells had reduced expressions of TCF7L1 and NE markers and increased expressions of androgen-responsive genes (Fig. 5E). Consistently, CSS-treated cells had increased TCF7L1 and NE markers and decreased androgen-responsive gene expressions, while in cells with WNT4-KD, levels of TCF7L1 and NE markers were reduced, and those of androgen-responsive genes were increased. (Fig. 5F). These data suggest that WNT4 stimulation is related to activation of TCF7L1-driven NED in PCa after ADT. We next knocked down TCF7L1 and further treated cells with the WNT4 protein, and found that TCF7L1-KD cells exhibited abolished expressions of WNT4-mediated IL-8 and CXCR2 (Fig. 5G, H), supporting that WNT4 upregulation associated with IL8/CXCR2 expression is TCF7L1-dependent in PCa cells. It was shown that targeting WNT3A signaling reduces β-catenin expression and may be considered to have therapeutic potential in NEPC [47]. We further investigated whether WNT3A can induce IL-8/CXCR2 expression in a TCF7L1-dependent manner by treating TCF7L1-KD cells with the WNT3A protein. We found that WNT3A could still upregulate IL-8 and CXCR2 expressions, regardless of whether TCF7L1 was knocked down in cells (Supplementary Fig. S1F). This result indicates that WNT3A may also mediate IL-8/CXCR2 expression, but through a TCF7L1-independent pathway. In addition, LNCaP and C4-2 cells treated with the WNT4 protein had increased NE marker protein levels and decreased androgen-responsive genes; however, cells with TCF7L1-KD did not show these effects (Fig. 5I). To test the effect of WNT4 on transcriptional activities of the *IL-8* and *TCF7L1* genes, results from the ChIP assay showed that TCF7L1-binding signals of the *IL-8* and *CXCR2* genes increased in cells in response to WNT4 protein treatment, whereas TCF7L1-KD decreased this effect (Supplementary Fig. S2C, D). Moreover, increased *IL-8* and *CXCR2* reporter activities were found in cells with WNT4 protein treatment; however, TCF7L1-KD attenuated the ability of WNT4 to upregulate *IL-8* and *CXCR2* reporter activities according to reporter assays (Supplementary Fig. S2E, F). These data are consistent with our hypothesis that WNT4 upregulation is associated with TCF7L1-mediated transcription of the *IL-8* and *CXCR2* genes.

### WNT4/TCF7L1 enhances cell migration and invasion of PCa cells

**Fig. 6:**
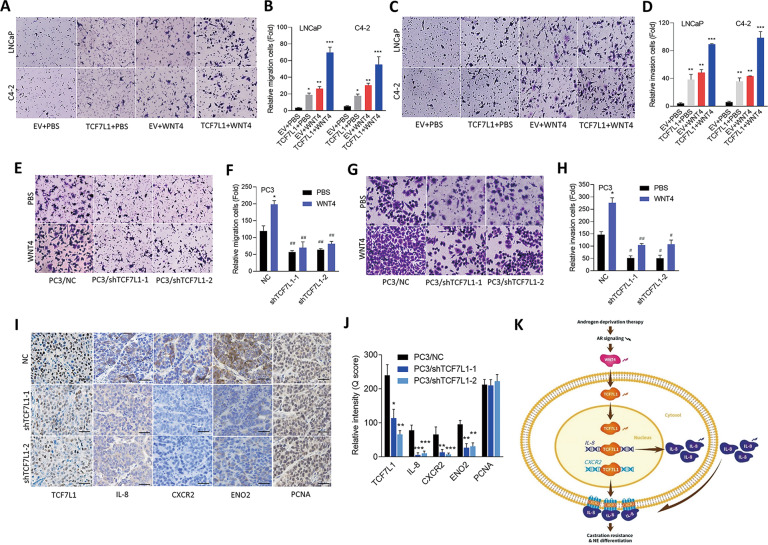
WNT4/TCF7L1 upregulate cell migration and the NED of PCa cells. **A–D** Cell migration (**A**, **B**) and invasion (**C**, **D**) assay of LNCaP and C4-2 cells with stable overexpression of TCF7L1 or an empty vector (EV) following 100 ng/ml WNT4 treatment for 24 h. * vs. the EV + PBS. Data are presented as the mean ± SEM, *n* = 3. *p < 0.05, **p < 0.01, ***p < 0.001; by a two-way ANOVA. **E–H** Cell migration (**E**, **F**) and invasion (**G**, **H**) assays of PC3 cells with overexpression of a nontarget control (NC) or TCF7L1 shRNA vector following 100 ng/ml WNT4 treatment for 24 h. * vs. PBS; ^#^ vs. the NC. Data are presented as the mean ± SEM, *n* = 3. *p < 0.05, **p < 0.01, ***p < 0.001; by a two-way ANOVA. **I**, **J** IHC staining and analysis of subcutaneous tumors with antibodies specific for TCF7L1, IL-8, CXCR2, ENO2, and PCNA in mice harboring PC3 cells expressing the NC or TCF7L1 shRNA vectors. Scale bars represent 100 µm. **K** Proposed model for androgen deprivation-induced WNT4/TCF7L1 signaling crosstalk with IL-8/CXCR2 signaling. ADT induced activation of the WNT4/TCF7L1 pathway, leading to upregulation of IL-8 and CXCR2. Overexpression of IL-8 and CXCR2 drives ADT resistance and NED of PCa.

In order to evaluate the contribution of WNT4/TCF7L1 to the malignant progression of human prostate adenocarcinomas, AR-positive C4-2 and LNCaP cells stably expressing TCF7L1 were treated with the WNT4 protein. The roles of WNT4/TCF7L1 in driving cell migration, invasion, and proliferation were further tested. Results showed that cells with TCF7L1 overexpression or WNT4 treatment had significantly increased cell migration and invasion compared to phosphate-buffered saline (PBS)-treated cells expressing an EV, while TCF7L1-expressing cells treated with WNT4 had the strongest cell migration and invasion abilities (Fig. 6A–D). These results confirmed the synergistic effect of WNT4 and TCF7L1 of increasing cell migration and invasion. Interestingly, we found that cells with TCF7L1 overexpression did not exhibit an increased proliferation rate even in cells treated with the WNT4 protein (Supplementary Fig. S3A, B), indicating that TCF7L1 mainly affects cell migration rather than cell proliferation. In addition, AR-negative PC3 cells with WNT4 protein treatment had increased cell migration and invasion capabilities according to cell migration and invasion assays; however, cells expressing TCF7L1-KD did not exhibit these effects (Fig. 6E–H). Notably, there were no significant changes in cell proliferation of PC3 cells expressing TCF7L1-KD, even in cells with WNT4 treatment (Supplementary Fig. S3C). These results were supported by further in vivo experiments. Mice that were administered subcutaneous injections of PC3 cells harboring TCF7L1-KD showed no changes in tumor growth rates or tumor weights compared to mice injected with cells carrying the NC shRNA (Supplementary Fig. S3D–F). To verify the ability of TCF7L1 to upregulate IL-8/CXCR2 and mediate cellular NED, tumors were harvested and examined for TCF7L1, IL-8, CXCR2, and NE and proliferation markers by IHC. Results showed that tumors from PC3 cells with TCF7L1-KD had decreased TCF7L1, IL-8, CXCR2, and NE marker expressions compared to NC-expressing cells, but there was no significant difference in cell proliferation markers (Fig. 6I, J), confirming that TCF7L1 is an important factor that induces IL-8/CXCR2-associated NED in PCa cells. These data suggest that WNT4/TCF7L1 promotes a variety of cell motility properties and the NED of PCa cells. Taken together, we demonstrated that inhibiting AR activities increased TCF7L1 expression, and induction of TCF7L1 through upregulation of WNT4 relieved activation of the oncogenic roles of IL-8 and CXCR2, subsequently leading to the development of malignant progression and the NED of PCa (Fig. 6K).

## Discussion

Evidence indicates that AR inhibition in PCa after ADT can activate the WNT signaling pathway [26, 48, 49]. Exploring the relationship between AR pathway inhibition and WNT transcriptional activity is important for patients with PCa who may require different management strategies for their disease [50]. We studied WNT ligands that are upregulated after ADT, which may be related to the progression of NED in PCa cells undergoing MDV3100 resistance. Indeed, ß-catenin and various WNT ligands, including WNT4, WNT5A, WNT7B, and WNT11, were shown to play roles in the development of ADT resistance in PCa cells [51–53]. In addition, it was reported that WNT7B and WNT11 can increase expressions of NE markers in PCa cells [24, 54]. Although secretion of WNT ligands is common in PCa after ADT treatment [7], each ligand may induce ADT resistance and NED progression through different signaling pathways. Our results demonstrated the role of WNT4 in upregulating TCF7L1-dependent NED and linked TCF7L1 to IL-8/CXCR4-driven NED of PCa.

Activation of WNT signaling is highly associated with NED due to their impacts on neurodevelopment [44]. Our data support the drug-resistance role of WNT4 in PCa. We indentified the regulatory mechanism underlying the impact of WNT4 in TCF7L1-driven IL-8/CXCR2 signaling on the NED of PCa after ADT. Our model was supported by ectopic WNT4 expression and the addition of exogenous WNT4. Both of these had indirect effects on WNT4 binding to secreted frizzled (FZD)-related protein (sFRP) family proteins or by replacing other endogenous WNT proteins with the opposite effect of binding to FZD receptors [55, 56]. We focused on WNT4 because it is more highly expressed in ADT-resistant cells. Our data confirmed that WNT4 gene silencing in MDV3100-resistant C4-2 and CSS-treated LNCaP cells reduced expressions of TCF7L1 and NE markers, which provides support for the link between endogenous WNT4 and TCF7L1 expressions in PCa cells under AR deprivation. Our study suggests that inhibition of AR-increased WNT4 upregulation activates TCF7L1-driven IL-8/CXCR2 signaling which is associated with PCa NED progression. We explored the regulatory mechanisms of TCF7L1 which link suppression of the AR pathway with upregulated WNT4 signaling, and defined the roles of TCF7L1 in this crosstalk leading to progression of NED PCa by upregulating IL-8 and CXCR2.

The relationship between cytokine signaling and PCa progression has been investigated for several years [57]. However, the connection between WNT signaling and cytokine responses in NED PCa cells is still unclear, and effective cytokine-targeted therapies for NED PCa are also still unavailable. IL-8 is known as a downstream effector of WNT signaling, which is essential for cancer progression, metastasis, and development of chemoresistance [58]. As ovarian tumor cells undergo the EMT, those cells activate WNT signaling to promote IL-8 expression and further reinforce an IL-8/CXCR2/WNT feedback loop [59]. The IL-8/CXCR2/WNT feedback loop was also observed in the development of chemoresistance and metastasis in estrogen receptor (ER)-positive breast cancer, in which WNT signaling is specifically mediated by WNT3A coupled with SNAI1/nuclear factor (NF)-κB signaling activation [60]. In PCa, we demonstrated that IL-8/CXCR2 signaling leads to interactions with WNT4/TCF7L1 in the NED progressive signaling pathway, thereby mediating the ability of aggressive phenotypes of PCa post-ADT. Our study provides a functional link between low AR activities and increased TCF7L1, which may be associated with WNT4 stimulation that contributes to TCF7L1-driven PCa NED progression. This supports low AR activity resulting in elevation of the WNT pathway at low androgen levels and in turn promoting castration-refractory and NED PCa progression [25, 26, 48, 49]. Given that IL-8/CXCR2 overexpression in NE cells enhances a high metastatic potential [36], we demonstrated that the activated WNT4/TCF7L1 pathway promotes NED and cytokine responses of PCa through upregulating IL-8/CXCR2 signaling.

In summary, our report supports the increased aggressiveness of PCa cells after ADT possibly being accompanied by cellular NED. We demonstrated that ADT may induce the upregulation of WNT4/TCF7L1 signaling, leading to the activation of IL-8/CXCR2 to drive the progression of NED. Inhibition of AR signaling is sufficient to increase WNT4 and TCF7L1 expressions, which implies a dominant biological function of WNT4/TCF7L1 for NED progression of PCa in the WNT signaling regulatory network. Our data provide a mechanism whereby high levels of TCF7L1 post-ADT result in increased IL-8 and CXCR2 levels through upregulation of WNT4, in turn leading to NED progression in PCa. A novel regulatory role for the candidate prostate tumor promoter, TCF7L1, was identified. We demonstrated that the interaction of TCF7L1 with the IL-8/CXCR2 signaling pathway requires the presence of components of WNT4 signaling. This could explain why crosstalk between the cytokine response and WNT signaling is frequently associated with NED PCa progression.

## Materials and methods

### Cell lines and cell culture

The human PCa LNCaP, C4-2, and PC3 cell lines were acquired from American Type Culture Collection (ATCC; Manassas, VA, USA). All cells were cultured in RPMI 1640 medium (Thermo-Fisher, Waltham, MA, USA) supplemented with 10% fetal bovine serum (FBS). The NE-like NE-1-8 cell line was purchased from ATCC and cultured in RPMI 1640 medium supplemented with 5% charcoal-stripped serum (CSS) and Dulbecco’s modified Eagle medium (DMEM) supplemented with 10% FBS, 0.005 mg/ml bovine insulin (Sigma–Aldrich, Darmstadt, Germany), and 10 nM dihydrotestosterone (DHT; Sigma–Aldrich). The LASCPC01 cell line was obtained from ATCC and was cultured in HITES medium which was composed by RPMI 1640 medium, 1× insulin-transferrin-selenite (ITS-G, Thermo-Fisher), 2 mM GlutaMAX (Thermo-Fisher), 10 μM hydrocortisone (Sigma–Aldrich), 10 μM β-estradiol (Sigma–Aldrich), 1× penicillin/streptomycin (Thermo-Fisher), and 5% FBS (Thermo-Fisher). The MDV3100-resistant C4-2-MDVR cell line is a viable cell line generated by growing C4-2 cells under selective pressure of 20 μM enzalutamide (MDV3100) for 6 months. For DHT treatment, cells were treated with 10 nM DHT (Sigma–Aldrich) for 24 h in 10% CSS-containing medium. All cell lines tested negative for mycoplasma contamination. For WNT recombinant protein treatment, cells were treated with 100 ng/ml of human WNT4 or WNT3A (R&D Systems, Minneapolis, MN, USA) for 48 h, in 10% CSS-containing medium. For the porcupine inhibitor IWP-O1 treatment, cells were treated with 1 μM IWP-O1 (Sigma–Aldrich) for 48 h in 10% FBS-containing medium. All cellular experiments were carried out within 20 passages to ensure consistency and uniformity.

### Real-time reverse-transcription quantitative polymerase chain reaction (RT-qPCR)

The experimental procedures are detailed in “Supplementary Information”.

### Immunohistochemical (IHC) staining

Clinical samples consisted of 11 independent SCPC tumors collected from Taipei Medical University-Wan Fang Hospital (Taipei, Taiwan). PCa TMA sections, including 12 normal prostatic epithelial samples, 77 primary prostate adenocarcinomas, and eight SCPCs, and CRPC TMA sections, including 25 CRPC patients, were provided by Duke University School of Medicine (Durham, NC, USA). Eighteen PCa samples from the same patients before and after ADT were collected from Taipei Medical University-Wan Fang Hospital (Taipei, Taiwan). Tissue samples were used in accordance with the U.S. Common Rule and the *Declaration of Helsinki*, and their use was approved by the Duke University School of Medicine Institutional Review Board (protocol ID: Pro00070193) and the Taipei Medical University Joint Institutional Review Board (approval no.: N202001017). IHC was performed using TCF7L1 (14519-1-AP; Proteintech, Rosemont, IL, USA) and CXCR2 (555932; BD Biosciences, San Jose, CA, USA) antibodies at respective 1:150 and 1:200 dilutions as described in “Supplementary Materials and Methods”.

### Cytokine array assay

A cytokine assay was performed using the human cytokine array kit (ARY005B, R&D Systems) following the protocol in the user’s manual as described in “Supplementary Materials and Methods”.

### Chromatin immunoprecipitation (ChIP) assay

The experimental procedures are detailed in “Supplementary Information”.

### Promoter reporter assay

The experimental procedures are detailed in “Supplementary Information”.

### Western blot analysis

The experimental procedures are detailed in “Supplementary Information”.

### Polymerase chain reaction (PCR)-array analysis

The experimental procedures are detailed in “Supplementary Information”.

### Migration and invasion assays

The experimental procedures are detailed in “Supplementary Information”.

### Cell proliferation assay

The experimental procedures are detailed in “Supplementary Information”.

### Tumorigenicity assays in mice

Animal work was performed in accordance with a protocol approved by the Taipei Medical University Animal Care and Use Committee (approval no.: LAC-2019-0465, Taipei, Taiwan). Five-week-old male nude mice (NLAC, Taipei, Taiwan) were randomly subcutaneous injected with TCF7L1 short hairpin (sh)RNA or nontarget control (NC) vector-transfected PC3 cells in 50% Matrigel^TM^ (BD Biosciences). The tumor volume was measured weekly with calipers, and the tumor volume was calculated using the following formula: tumor volume = (4/3) × (L/2) (W/2)^2^, where L is the length and W the width. The results are presented as the mean ± standard error (SE) for each experimental group. At the end of the 40th day, tumors of mice injected with PC3 cells expressing TCF7L1-knockdown (KD) or the control vector were collected, and the weights were measured. Tumors were fixed in 4% paraformaldehyde (PFA) in PBS, and IHC staining was performed.

### Statistical analysis

Experimental results were collected and plotted by GraphPad Prism v8.0 (GraphPad Software, San Diego, CA, USA). Data from each group were compared using an analysis of variance (ANOVA) and Dunnett’s test for a post-hoc analysis compared to the control groups. All data are presented as the mean ± standard error of the mean (SEM). Data groups significantly differing from the control were appropriately labeled for comparisons among three or more groups. A log-rank test was used for the survival curve analysis. *p* values of <0.05 were considered statistically significant.

## Supplementary information

Supplementary Information
