# Supplementary material for: TCF7L1 regulates cytokine response and neuroendocrine differentiation of prostate cancer
Source: Oncogenesis. 2021 Nov 20;10(11):81. doi: 10.1038/s41389-021-00371-6 (PMC8604986; doi:10.1038/s41389-021-00371-6)
Supplement: Supplementary file 1 — Supplementary Information [file 41389_2021_371_MOESM1_ESM.docx]

**Supplementary Information**

**Supplementary** **Materials and Methods**

**Reagents and constructs**

TCF7L1 and WNT4 overexpression was generated by establishing a pCDH-CMV-MCS-EF1-Puro vector (System Biosciences, Palo Alto, CA, USA) encoding TCF7L1 and WNT4 cDNA; an empty vector (EV) was used as a control. Knockdown of TCF7L1 was generated by infection with a recombinant lentivirus encoding human TCF7L1 shRNA (RNAi Core Lab, Taipei, Taiwan); a non-target control (NC) pLKO_TRC005-Puro vector was used as a control. Small interfering (si)RNAs (NC and siWNT4) were obtained from ON-TARGETplus SMARTpool siRNA (D-001810-10 and 008659-03-0005, Thermo Scientific Dharmacon, Waltham, MA, USA). Regulatory sequence reporters of the *CXCR2* and *IL-8* genes were constructed using the pGreenFire reporter (System Biosciences), and a Site-Directed Mutagenesis System kit (Invitrogen, Waltham, MA, USA) was used for response element mutations. All primers used to generate these constructs are listed in Supplementary Table S1. All constructs were verified by a DNA sequence analysis.

**Dataset analysis**

Enriched genes in CXCR2^+^ neuroendocrine (NE) populations through an RNA-Sequencing (RNA-Seq) analysis were found using transcriptome profiling data downloaded from GEO (GSE114326). The *TCF7L1* gene is upregulated in CXCR2^+^ NE populations compared to CXCR2^-^ luminal populations purified from primary fresh human prostate cancer (PCa) tissues based on the CXCR2 cell-surface receptor [^2^](#_ENREF_2). Totals of 151 primary and 19 metastatic samples in the Taylor prostate cancer dataset [^5^](#_ENREF_5) were accessed under Memorial Sloan Kettering Cancer Center (MSKCC) Institutional Review Board approval. In total, 414 primary PCa samples of patients treated with a radical prostatectomy in the public human PCa databases from TCGA were accessed under National Cancer Institute, National Institutes of Health Review Board approval. Expression data (and resulting z-scores) were log2-normalized. Gene sets of neuronal development-responsive signaling (Gene Ontology (GO) and KEGG), CXCR2^+^ NE-response [^2^](#_ENREF_2), CRPC-NE-response [^1^](#_ENREF_1), and androgen response (GO, PID, Wang [^6^](#_ENREF_6), and Nelson [^3^](#_ENREF_3)) gene signatures were used to determine correlations with TCF7L1 levels by a GSEA and z-score analysis. The number of permutations was set to 1000, and the permutation type was set to “phenotype”. A normalized enrichment score (NES) and false discovery rate (FDR) were calculated by the GSEA program. The method for determining cutoffs was pre-decided using half of the number of patients. Tumors were mean-stratified by TCF7L1 expression, and the mean expression of each of these genes was determined in each group. The RNA-Seq dataset of paired PCa samples pre- and post-ADT was downloaded from GEO (GSE48403). Expression data were log2-normalized.

**Real-time reverse-transcription quantitative polymerase chain reaction (RT-qPCR)**

An RNeasy Midi Kit (Qiagen, Redwood City, CA, USA) was used for total RNA isolation. For the RT-PCR, 1 µg of total RNA was used with a one-step real-time RT-PCR kit (Bio-Rad, Hercules, CA, USA). Reactions for all primer pairs were performed using a thermocycler at an initial temperature of 95 °C for 10 min, followed by 40 cycles of 95 °C for 15 s and 60 °C for 1 min. The amplification step used the SYBR green PCR master mix (Applied Biosystems, Waltham, MA, USA). All reactions were normalized to human *GAPDH* and run in triplicate. All primers used for the PCR are listed in Supplementary Table S2.

**Immunohistochemical (IHC) staining**

Clinical samples consisted of 11 independent small-cell NE PCa (SCPC) tumors collected from Taipei Medical University-Wan Fang Hospital (Taipei, Taiwan). PCa tissue microarray (TMA) sections, including 14 normal prostatic epithelial samples, 77 primary prostate adenocarcinomas, and eight SCPCs; and castration-resistant PCa (CRPC) TMA sections, including 25 CRPC patients, were provided by Duke University School of Medicine (Durham, NC, USA). Eighteen PCa samples from the same patients before and after ADT were collected from Taipei Medical University-Wan Fang Hospital (Taipei, Taiwan). Tissue samples were used in accordance with the U.S. Common Rule and *Declaration of Helsinki*, and their use was approved by the Duke University School of Medicine Institutional Review Board (protocol ID: Pro00070193) and the Taipei Medical University Joint Institutional Review Board (approval no.: N202001017). IHC was performed using TCF7L1 (14519-1-AP; Proteintech, Rosemont, IL, USA) and CXCR2 (555932; BD Biosciences, San Jose, CA, USA) antibodies at respective 1:150 and 1:200 dilutions. In general, unstained sections were deparaffinized and rehydrated. Antigen retrieval was performed using Target Antigen Retrieval Solution (Dako, Santa Clara, CA, USA) and autoclaved for 10 min. Endogenous peroxidase was blocked using a 3% hydrogen peroxide solution. All sections were blocked with Cyto Q Background Buster Reagent (Innovex BioSciences, Richmond, CA, USA). Primary antibodies were incubated overnight at 4 °C in Antibody Diluent with Background Reducing Components (Dako). A secondary antibody, 1:250 horseradish peroxidase (HRP)-labeled anti-mouse/rabbit antibody (Vector laboratories, Burlingame, CA, USA), was incubated at room temperature for 30 min, and bound peroxidase was detected using an ABC Peroxidase Kit (Vector Laboratories) and DAB (DAKO). All IHC slides were counterstained with hematoxylin. For the histomorphometric analysis of tissue sections, microscopic images were examined under 200× magnification using an Axioplan microscopic system (Zeiss, White Plains, NY, USA). The intensity was denoted as 0 (negative), 1+ (weakly positive), 2+ (moderately positive), and 3+ (strongly positive). Q-score values (range 0~300) were calculated using the following formula: [1 × (% cells with intensity of 1+) + 2 × (% cells with intensity of 2+) + 3 × (% cells with intensity of 3+)]. Associations between the relative intensities of prostatic tumors and IHC scores were determined by correlation XY analyses in GraphPad Prism or a two-tailed Student’s *t*-test.

**Cytokine array assay**

A cytokine assay was performed using the human cytokine array kit (ARY005B, R&D Systems, Minneapolis, MN, USA) following the protocol in the user's manual. Supernatant at 700 μL or 300 μg of cell lysate from PC3 cells expressing TCF7L1 shRNA or an NC vector was mixed with 500 μL of array buffer 4 and 15 μL of an antibody cocktail, and the volume was adjusted to 1.5 mL with array buffer 5. The sample mixture was incubated at room temperature for 1 h prior to being loaded onto pre-blocked array membranes and incubated at 4 °C overnight. Membranes were washed with wash buffer and then incubated with streptavidin-HRP for 30 min. Antibody-binding blots were determined by incubation with a chemi reagent mix and photographed with an imager.

**Chromatin immunoprecipitation (ChIP)** **assay**

ChIP assays were performed using the EZ magna ChIP A kit (Merck Millipore, Temecula, CA, USA) with a modified protocol. PC3 cells (10^7^) expressing the NC or TCF7L1 shRNA vector and LNCaP cells (10^7^) expressing the EV or TCF7L1 cDNA vector were treated with or without WNT4 (100 ng/ml) for 48 h and cross-linked with 1% formaldehyde at room temperature for 15 min. Fixation was quenched with glycine, and cells were washed twice with cold phosphate-buffered saline (PBS) containing a complete protease inhibitor (Roche, Little Falls, NJ, USA). Cell pellets were resuspended in cell lysis buffer and incubated on ice for 15 min. Nuclei were collected by centrifugation at 10^4^ rpm and 4 °C for 10 min and resuspended in nuclear lysis buffer. Chromatin was sheared using a sonicator (Branson Digital Sonifier SFX 250; Emerson, St. Louis, MO, USA) with a microtip in a 20-s burst followed by 1 min of cooling on ice for a total sonication time of 5 min/sample. This procedure resulted in DNA fragment sizes of 100~300 bp. Sheared chromatin was divided to perform immunoprecipitation (IP) with a rabbit immunoglobulin G (IgG) antibody (Santa Cruz, CA, USA) or primary antibody at 4 °C overnight. IP, washing, elution, reverse cross‐linking, and DNA purification steps were performed according to Merck Millipore’s protocol. A qPCR was performed in triplicate with 2 μl of eluted chromatin. ChIP antibodies and PCR primers are listed in Supplementary Table S3. TCF7L1-binding sites were located on the *CXCR2* regulatory sequence using a genome-wide binding profile of TCF7L1 in human embryonic stem cells (GSE80331) [^4^](#_ENREF_4) and analyzed at GRCh37 from the Genome browser gateway (UCSC Genomics Institute, Santa Cruz).

**Promoter reporter assay**

Promoter function was analyzed using fluorescence-activated cell sorting (FACS), and relative median fluorescent intensity (MFI) values were measured using FACS (BD Biosciences, San Jose, CA, USA) with FACS Diva software (BD Biosciences), normalized to the value of the vehicle. The MFI was determined from the first fluorescent peak. LNCaP and PC3 cells in 12-well plates (5×10^4^ cells/well) were transiently transfected with 1 µg of the *CXCR2*- or *IL-8*-GFP reporter containing individual wild-type (WT) and a mutant of the putative TCF7L1-binding sites. Cells were treated with or without WNT4 (100 ng/ml) for 48 h. The GFP MFI value was measured by FACS using FACSDiva software (BD Biosciences) and was normalized to the value of the vehicle. Three independent experiments were conducted in triplicate.

**Western blot analysis**

Cells at 2×10^6^ cells/treatment were inoculated into 10-cm dishes and treated appropriately. When treatment was completed, cells were washed with PBS, mixed with RIPA buffer, and incubated on ice for 10 min. The concentration of total soluble proteins from each treatment was quantified using the Bradford assay (Bio-Rad, Hercules, CA, USA) after debris removal by centrifugation at 16,000 ×*g* for 10 min. Then, 20 μg of crude protein was separated by sodium dodecylsulfate polyacrylamide gel electrophoresis (SDS-PAGE), transferred to a 0.22-μm polyvinylidene difluoride membrane, blocked by 1% BSA/TBST (TBS buffer with 0.1% Tween-20) at room temperature for 1 h, and sequentially stained with an appropriate 1^st^ antibody at 4 °C overnight and a 2^nd^ antibody at room temperature for 1 h. Primary antibodies and the dilutions used are listed in Supplementary Table S4. Finally, membranes were soaked in enhanced chemiluminescence (ECL) reagent (Merck Millipore) and used to detect stained protein blots with an imager (Cytiva Amersham Imager 600, Washington, DC, USA).

**PCR-array analysis**

Measurement of WNT signaling in C4-2-MDVR and parental C4-2 cells was carried out with a human WNT signaling RT^2^ Profiler PCR Array (PAHS-043Y; Qiagen, Venlo, the Netherlands) which was based on an RT-qPCR. Cells were cultured in FBS-containing medium and washed with PBS, and their mRNA was extracted with an rSYNC RNA isolation kit (Geneaid, New Taipei City, Taiwan). Total RNA from each sample was quantified using a NanoDrop Microvolume Spectrophotometer (Thermo-Fisher, Waltham, MA, USA), and 1 μg of RNA was converted into cDNA by an iScript cDNA synthesis kit (Bio-Rad). cDNA products at 1 μL were mixed with 10 μL iTaq™ Universal SYBR^®^ Green mix, and 9 μL of RNase-free water, and then loaded for a PCR array followed by a real-time PCR assay using the StepOne^TM^ real-time PCR system (Thermo-Fisher). The PCR program was as listed below in which step 2 was repeated 45 times.

**Migration and invasion assay**

LNCaP and C4-2 cells expressing an EV or TCF7L1 cDNA vector and PC3 cells expressing an NC or TCF7L1 shRNA vector were treated with or without 100 ng/ml WNT4 protein for 24 h. BD Matrigel^TM^ for the invasion assay was purchased from BD Biosciences. Matrigel-coated transwell dishes were prepared by adding 200 μl of CSS-containing medium diluted with Matrigel. In total, 2.5×10^5^ cells/well in CSS-containing medium were plated above the Matrigel. The lower chamber was filled with 600 μl FBS-containing medium with 100 ng/ml WNT4 added. After 24 h, cells that had invaded the Matrigel-coated transwells in response to WNT4 were fixed and stained with a 0.5% crystal violet fixative solution for 15 min. Invaded cells on the underside of the membrane were counted and quantified in five medium-power fields for each replicate in triplicate. The migration assay used transwells without Matrigel, and cells were fixed and stained as described for the invasion assay.

**Cell proliferation assay**

LNCaP and C4-2 cells expressing an EV or TCF7L1 cDNA vector or PC3 cells expressing a TCF7L1-knockdown (KD) or NC vector were seeded at a density of 2000 cells/well in 96-well plates. Cells were treated with the WNT4 protein (100 ng/ml) in FBS-containing medium for 5 days. Multiple wells were assessed using a Cell Proliferation Assay Kit (Promega, Madison, WI, USA) according to the manufacturer’s protocol. The absorbance was quantified at a wavelength of OD 550 nm on a plate reader at each time point and then averaged.

**
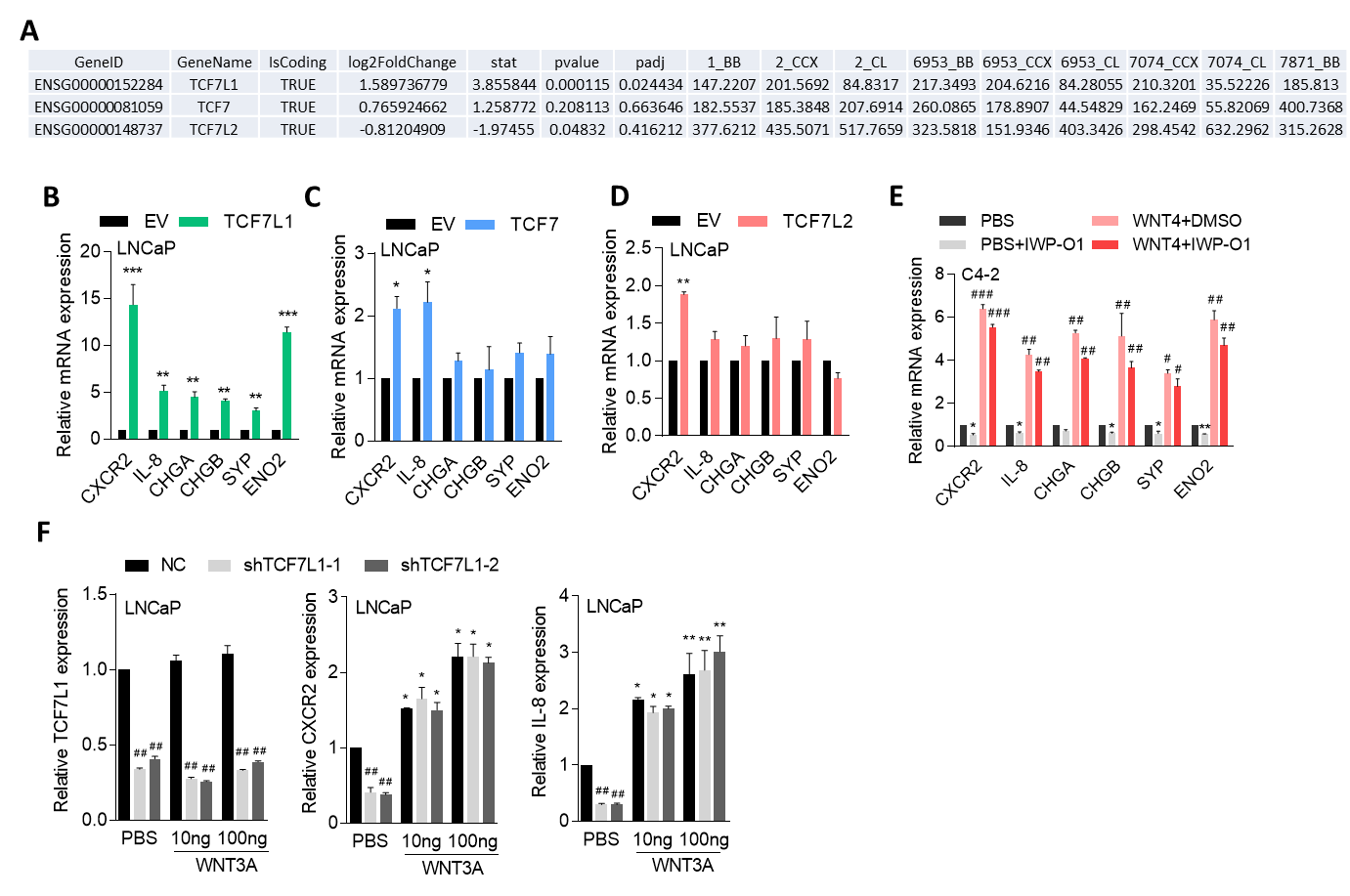
Supplementary Figures**

**Supplementary Fig. S1: TCF7L1 regulates IL-8/CXCR2-associated NED by WNT4. A** The log2-fold change values and *p* values of the *TCF7L1, TCF7,* and *TCF7L2* genes in GSE114326 RNA-Seq data. **B-D** RT-qPCR showing CXCR2, IL-8, and NE marker mRNA levels in LNCaP cells transiently expressing an empty vector (EV), TCF7L1 (**B**), TCF7 (**C**), or TCF7L2 (**D**) cDNA vector. **E** RT-qPCR showing CXCR2, IL-8, and NE marker mRNA levels in C4-2 cells treated with 100 ng/ml WNT4 protein for 24 h after pretreatment with 1 μM IWP-O1 for 24 h. **F** mRNA levels of TCF7L1, CXCR2, and IL-8 in LNCaP cells expressing the NC or TCF7L1 shRNA vector following increased WNT3A protein treatment for 48 h, by an RT-qPCR analysis. * vs. PBS; ^#^ vs. the NC. Data from the quantification of mRNA are presented as the mean ± SEM, *n*=3 per group; by a two-way ANOVA.

**
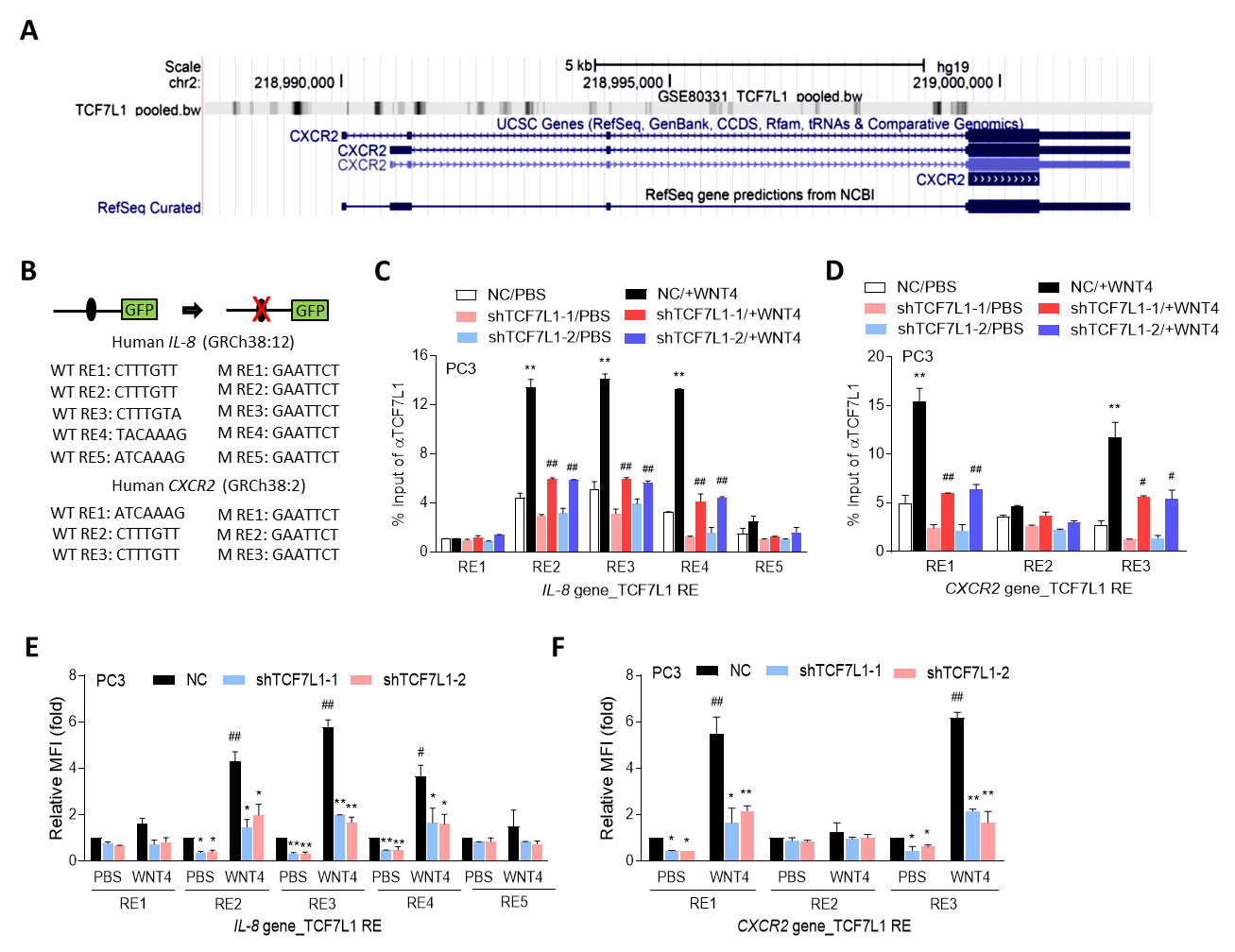
**

**Supplementary Fig. S2: TCF7L1 upregulates *IL-8* and *CXCR2* through increased WNT4.** **A** ChIP-sequence analysis of detected DNA-binding sites for nuclear TCF7L1 using a genome-wide binding profile of TCF7L1 in human embryonic stem cells [^4^](#_ENREF_4). Schematic of the predicted TCF7L1-responsive elements in regulatory sequences of the human *CXCR2* gene as labeled as black set lines in the tracks. **B** Schematic and sequence of the predicted TCF7L1 response element (RE) and the introduced binding site mutant in the regulatory sequence reporter constructs of human *IL-8* and *CXCR2*. **C, D** ChIP assays of PC3 cells expressing a non-target control (NC) or TCF7L1 shRNA vector following 100 ng/ml WNT4 protein treatment for 48 h with antibodies against TCF7L1 by a pair of primers recognizing indicated TCF7L1 RE sites on the *IL-8* (**C**) and *CXCR2* (**D**) regulatory sequences. Enrichment is given as a percentage of the total input and then normalized to IgG. * vs. PBS; ^#^ vs. the NC. **E, F** Medium fluorescent intensities (MFIs) of *IL-8* and *CXCR2* green fluorescent protein (GFP)-reporters in PC3 cells stably transfected with the NC or TCF7L1 shRNA vector following 100 ng/ml WNT4 protein treatment for 48 h. Quantification of ChIP data, and MFIs are presented as the mean ± SEM of three independent experiments. Significance was determined by Student’s *t*-test. * *p*<0.05, ** *p*<0.01, *** *p*<0.001.


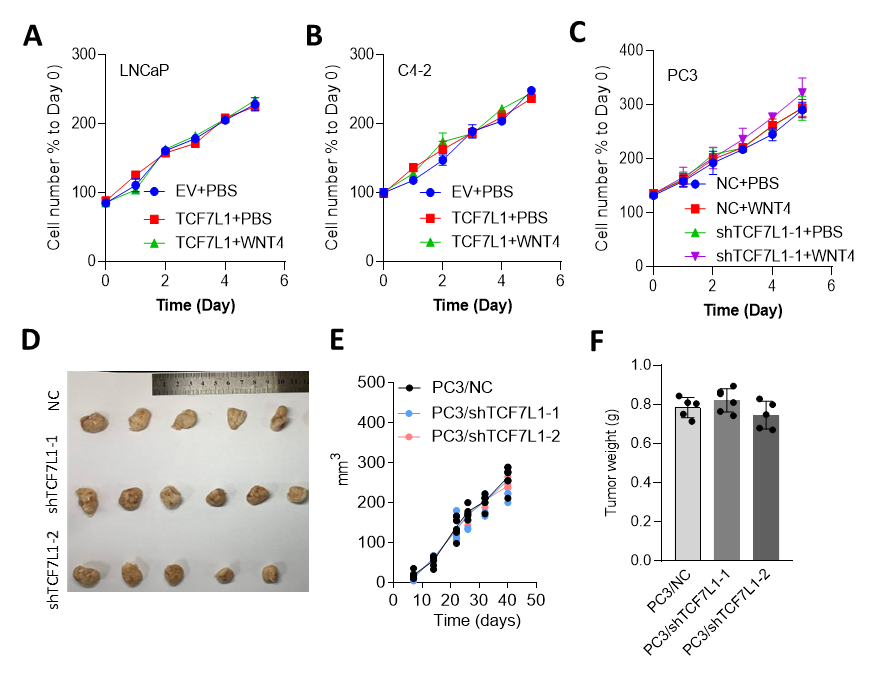


**Supplementary Fig. S3: WNT4/TCF7L1 upregulation did not affect cell proliferation of prostate cancer cells. A, B** Cell proliferation assay of LNCaP (**A**) and C4-2 (**B**) cells with empty vector (EV) or TCF7L1 cDNA vector overexpression following 100 ng/ml WNT4 treatment for 5 days. **C** Cell proliferation assay of PC3 cells with non-target control (NC) or TCF7L1 shRNA vector overexpression following 100 ng/ml WNT4 treatment for 5 days. Data are presented as the mean ± SEM, *n*=3. * *p*<0.05, ** *p*<0.01, *** *p*<0.001. **D, E** Tumor growth analysis using a xenograft mouse model with subcutaneous inoculation of PC3 cells stably expressing the NC (*n*=5), TCF7L1 (*n*=6), or TCF7L1-2 (*n*=5) shRNA vector in nude mice. Images of tumors from (**D**) at the end of day 40. **F** Quantitation of tumor weights of tumors from (**D**). Data are presented as the mean ± SEM. * *p*<0.05, ** *p*<0.01, *** *p*<0.001, **** *p*<0.0001.

**Supplementary Tables**

**Supplementary Table S1.** **Primer sequences of the promoter reporter constructs.** Primer sequences of the wild-type (WT) TCF7L1-binding elements of human *IL-8* and *CXCR2* promoter reporter (WT IL-8/RE1~5 and WT CXCR2/RE1~3) and the TCF7L1-binding element mutants of the human *IL-8* and *CXCR2* promoter reporter (M IL-8/RE1~5 and M CXCR2/RE1~3) are listed 5’-3’. WT, wild-type; M, mutant; F, forward; R, reverse).

| Promoter reporter | Sequence |
| --- | --- |
| WT IL-8/RE1 F | TGGGAAAAGTAGAGGCCATCT |
| WT IL-8/RE1 R | TTGCAGTGAGCCGAGATAGC |
| WT IL-8/RE2 F | CTCCTGAACTCGTGATCCGC |
| WT IL-8/RE2 R | TGATGAGCAGAGACTAGCACC |
| WT IL-8/RE3 F | TGTGTAGCGTGGCACTTTAGT |
| WT IL-8/RE3 R | GACTAAATGGGCTTAGGCGGA |
| WT IL-8/RE4 F | TGGGCCCATGAGAAGAGAATG |
| WT IL-8/RE4 R | TCCCTAACGGTTGCCTTTGT |
| WT IL-8/RE5 F | CAAATTGTGGAGCTTCAGTATTTTAAA |
| WT IL-8/RE5 R | TGATGGCCCATCCCCTCA |
| WT CXCR2/RE1 F | GGTTTGCCCTAGACCCTGTA |
| WT CXCR2/RE1 R | CGGTTATGTCCCCTTCAAGC |
| WT CXCR2/RE2 F | AAGTTCTCCAAGTCCCCACC |
| WT CXCR2/RE2 R | GCATAAATCTCACAGGGCCT |
| WT CXCR2/RE3 F | GCAAGGTCAGGGAAGCTTTC |
| WT CXCR2/RE3 R | TCAGAGCTTAAAGACAAGGCA |
| M IL-8/RE1 F | GCCATCTTTGTGAATTCTCTTTCTTTTTTAGA |
| M IL-8/RE1 R | TCTAAAAAAGAAAGAGAATTCACAAAGATGGC |
| M IL-8/RE2 F | GCGCCCGGCCGTGAATTCTCTTTCTTGAAC |
| M IL-8/RE2 R | GTTCAAGAAAGAGAATTCACGGCCGGGGCGC |
| M IL-8/RE3 F | GCGCCCGGCCGTGAATTCTCTTTCTTGAAC |
| M IL-8/RE3 R | GTTCAAGAAAGAGAATTCACGGCCGGGCGC |
| M IL-8/RE4 F | GGCTCACCATAAAGAATTCGGCAACCGTTA |
| M IL-8/RE4 R | TAACGGTTGCCGAATTCTTTATGGTGAGCC |
| M IL-8/RE5 F | TTATTTTAAAGAATTCAGAAAACTTTCGTC |
| M IL-8/RE5 R | CAGCAAAGTTTTCTGAATTCTTTAAAATAA |
| M CXCR2/RE1 F | CTCTCCAAGTGAGAATTCAGGTTTGCCCTAG |
| M CXCR2/RE1 R | CTAGGGCAAACCTGAATTCTCACTTGGAGAG |
| M CXCR2/RE2 F | TTGTTACCTAGATAGAATTCTTTTTTCATTGTGTT |
| M CXCR2/RE2 R | AACACAATGAAAAAAGAATTCTATACTAGGTAACAA |
| M CXCR2/RE3 F | ATTTCTTGGGGAATTCGTTCATTTCTTTT |
| M CXCR2/RE3 R | AAAAGAAATGAACGAATTCCCCAAGAAAT |

**Supplementary Table S2. RT-qPCR primer sequences.** Sequences of the primers used in real-time qPCR assays in this study are listed 5’-3’. F, forward; R, reverse; m, murine.

| Gene | Primer sequence | Gene | Primer sequence |
| --- | --- | --- | --- |
| *CXCR2* F | CTCAACCCCCTCATCTACGC | *CHGB* F | GCCACGTGCCTATTTCATGT |
| *CXCR2* R | GTGGAAGTGTGCCCTGAAGA | *CHGB* R | GCTCCTTCCTCACCGTAGTT |
| *TCF7* F | CGGGACAACTACGGGAAGAA | *SYP* F | GGCTTTGTGAAGGTGCTGC |
| *TCF7* R | TCCGGGTAAGTACCGAATGC | *SYP* R | CACTCTCGGTCTTGTTGGCAC |
| *TCF7L1* F | GGAAGAAAGTGGCACAACCTG | *ENO2* F | TCAGGGACTACCTGTGGTCT |
| *TCF7L1* R | GCTGACCAGGTTGGGTAGAG | *ENO2* R | TTCCACTGCCGCTCAATAC |
| *TCF7L2* F | CGGGAGAGACCAATGAACACA | *NKX3-1* F | CAGAGACCGAGCCAGAAACG |
| *TCF7L2* R | GGCCGCACCAGTTATTCTGT | *NKX3-1* R | CTGAGTGTGGGAGAAGGCAG |
| *IL-8* F | GAGAGTGATTGAGAGTGGACCA | *KLK3* F | TTTCCAATGACGTCTGTGCG |
| *IL-8* R | CACAACCCTCTGCACCCAGTTT | *KLK3* R | CCAGAATCACCCGAGCAGG |
| *CHGA* F | ACTGAAGGAGCTCCAAGACC | *GAPDH* F | CCAGTAGAGGCAGGGATGAT |
| *CHGA* R | TCTGCCTCCTTGGAATCCTC | *GAPDH* R | CTTTCATTGTCTTTTCCGCC |
| *WNT1* F | GGCCGATGGTGGGGTATTG | *WNT2* F | AGGATGCCAGAGCCCTGATGAA |
| *WNT1* R | GGAACTGCCACTTGCACTC | *WNT2* R | AGCCAGCATGTCCTGAGAGTAC |
| *WNT2B* F | CTAGGTCTTGCCTGCCTTCT | *WNT3A* F | ACAAAGCTACCAGGGAGTCG |
| *WNT2B* R | GGCTCACCAAACCAGGGATA | *WNT3A* R | TCCCACCAAACTCGATGTCC |
| *WNT4* F | GAGCAACTGGCTGTACCTGG | *WNT5A* F | ACTATGGCTACCGCTTTGCC |
| *WNT4* R | GGAACTGGTACTGGCACTCC | *WNT5A* R | CCAGGTTGTACACCGTCCTG |
| *WNT5B* F | CAACCAGTTCAAGAGCGTGC | *WNT6* F | TCGAGGCTCTTTATGGACGC |
| *WNT5B* R | GTACTGGTCCACGATCTCCG | *WNT6* R | CCACCTCGCGAAATGGAGG |
| *WNT7A* F | CATGGTCTACCTCCGGATCG | *WNT7B* F | GTCCTGTACGTGAAGCTCGG |
| *WNT7A* R | AAACTGACACTCGTCCAGGC | *WNT7B* R | CGGAACTGGTACTGGCACTC |
| *WNT8A* F | CCTTTCCCAACCCTCACTCC | *WNT8B* F | CCAAGCAGTTTGTCGATGCC |
| *WNT8A* R | ACTGGAACTTGCACTCCTCG | *WNT8B* R | TTTCATGGTGCCCTTCACCG |
| *WNT9A* F | GCGCGGCAAGATGCTGGATG | *WNT9B* F | CTTGAGCGGCGCGAGGAGATG |
| *WNT9A* R | CTGGGAACTCAGCCCTTGCAGG | *WNT9B* R | CCAGACTGGCTTGCTGGGCAG |
| *WNT10A* F | CATCCACGAATGCCAACACC | *WNT10B* F | CATCCTCAAGCGCGGTTTC |
| *WNT10A* R | CGCTCTCTCGGAAACCTCTG | *WNT10B* R | AAACTCTTGCCTCGGGACAG |
| *WNT11* F | TTGACCTGGAGAGAGGGACC | *WNT16* F | CAAGGAAACTGGATGTGGTTGG |
| *WNT11* R | CATGAGGAGCCCGTAGCTG | *WNT16* R | TGCAGTTCCATCTCTCGTGTC |

**Supplementary Table S3.** **ChIP antibodies and primer sequences.** Source and dilution of each antibody and the sequences (5’-3’) of each primer used for ChIP in this study are listed.

| ChIP antibodies | | | | | |
| --- | --- | --- | --- | --- | --- |
| Primary antibody | Species | | Clonality | Source | Dilution |
| TCF7L1 | Rabbit | | Polyclonal | Santa Cruz (sc-67022) | 1/50 |
| Acety-H3 | Rabbit | | Monoclonal | Novus (NB300-221) | 1/50 |
| IgG | Rabbit | |  | Santa Cruz (sc-2027) | 1/50 |
| ChIP primers | | | | | |
| Site | | Sequence | | | |
| IL-8/RE1 F | | TGGGAAAAGTAGAGGCCATCT | | | |
| IL-8/RE1 R | | TTGCAGTGAGCCGAGATAGC | | | |
| IL-8/RE2 F | | CTCCTGAACTCGTGATCCGC | | | |
| IL-8/RE2 R | | TGATGAGCAGAGACTAGCACC | | | |
| IL-8/RE3 F | | TGTGTAGCGTGGCACTTTAGT | | | |
| IL-8/RE3 R | | GACTAAATGGGCTTAGGCGGA | | | |
| IL-8/RE4 F | | TGGGCCCATGAGAAGAGAATG | | | |
| IL-8/RE4 R | | TCCCTAACGGTTGCCTTTGT | | | |
| IL-8/RE5 F | | CAAATTGTGGAGCTTCAGTATTTTAAA | | | |
| IL-8/RE5 R | | TGATGGCCCATCCCCTCA | | | |
| CXCR2/RE1 F | | GGTTTGCCCTAGACCCTGTA | | | |
| CXCR2/RE1 R | | CGGTTATGTCCCCTTCAAGC | | | |
| CXCR2/RE2 F | | AAGTTCTCCAAGTCCCCACC | | | |
| CXCR2/RE2 R | | GCATAAATCTCACAGGGCCT | | | |
| CXCR2/RE3 F | | GCAAGGTCAGGGAAGCTTTC | | | |
| CXCR2/RE3 R | | TCAGAGCTTAAAGACAAGGCA | | | |

**Supplementary Table S4.** **Western blotting antibodies.** Source and dilution of each antibody used for Western blotting in this study are listed.

| Primary antibody | Clonality | Source  (catalog no.) | Dilution | Secondary antibody | Source | Dilution |
| --- | --- | --- | --- | --- | --- | --- |
| TCF7L1 | Polyclonal | Abcam (ab86175) | 1/1000 | anti-rabbit IgG | Jackson Labs | 1/5000 |
| IL-8 | Polyclonal | Abcam (ab52612) | 1/1000 | anti-rabbit IgG | Jackson Labs | 1/5000 |
| CXCR2 | Polyclonal | Abcam (ab217314) | 1/1000 | anti-rabbit IgG | Jackson Labs | 1/5000 |
| CHGA | Monoclonal | Santa Cruz  (sc-393941 ) | 1/100 | anti-mouse IgG | Jackson Labs | 1/5000 |
| ENO2 | Monoclonal | Santa Cruz  (sc-21738) | 1/100 | anti-rabbit IgG | Jackson Labs | 1/5000 |
| SYP | Monoclonal | Abcam (ab32127) | 1/1000 | anti-rabbit IgG | Jackson Labs | 1/5000 |
| KLK3 | Monoclonal | Santa Cruz  (sc-7316) | 1/200 | anti-mouse IgG | Jackson Labs | 1/5000 |
| NKX3-1 | Monoclonal | Thermo-Fisher (MA5-15618) | 1/2000 | anti-mouse IgG | Jackson Labs | 1/5000 |
| β-actin | Polyclonal | GeneTex (GTX109639) | 1/1000 | anti-rabbit IgG | Jackson Labs | 1/20000 |

**References**

1 Beltran H, Prandi D, Mosquera JM, Benelli M, Puca L, Cyrta J *et al* (2016). Divergent clonal evolution of castration-resistant neuroendocrine prostate cancer. *Nat Med* **22:** 298-305.

2 Li Y, He Y, Butler W, Xu L, Chang Y, Lei K *et al* (2019). Targeting cellular heterogeneity with CXCR2 blockade for the treatment of therapy-resistant prostate cancer. *Sci Transl Med* **11**.

3 Nelson PS, Clegg N, Arnold H, Ferguson C, Bonham M, White J *et al* (2002). The program of androgen-responsive genes in neoplastic prostate epithelium. *Proc Natl Acad Sci U S A* **99:** 11890-11895.

4 Sierra RA, Hoverter NP, Ramirez RN, Vuong LM, Mortazavi A, Merrill BJ *et al* (2018). TCF7L1 suppresses primitive streak gene expression to support human embryonic stem cell pluripotency. *Development* **145**.

5 Taylor BS, Schultz N, Hieronymus H, Gopalan A, Xiao Y, Carver BS *et al* (2010). Integrative genomic profiling of human prostate cancer. *Cancer Cell* **18:** 11-22.

6 Wang G, Jones SJ, Marra MA, Sadar MD (2006). Identification of genes targeted by the androgen and PKA signaling pathways in prostate cancer cells. *Oncogene* **25:** 7311-7323.
